# The ferroxidases are critical for Fe(II) oxidation in xylem to ensure a healthy Fe allocation in *Arabidopsis thaliana*

**DOI:** 10.3389/fpls.2022.958984

**Published:** 2022-08-17

**Authors:** Qing-Yang Zhu, Yun Wang, Xing-Xing Liu, Jia-Yuan Ye, Miao Zhou, Xiang-Ting Jing, Wen-Xin Du, Wei-Jie Hu, Chao He, Ya-Xin Zhu, Chong-Wei Jin

**Affiliations:** ^1^College of Natural Resources and Environmental Science, Zhejiang University, Hangzhou, China; ^2^Planting Technology Extension Center of Dongyang, Jinhua, China

**Keywords:** xylem, Fe long-distance transport, Fe(II) oxidation, ferroxidase, Fe(III)-citrate

## Abstract

The long-distance transport of iron (Fe) in the xylem is critical for maintaining systemic Fe homeostasis in plants. The loading form of Fe(II) into the xylem and the long-distance translocation form of Fe(III)–citrate have been identified, but how Fe(II) is oxidized to Fe(III) in the xylem remains unknown. Here, we showed that the cell wall-resided ferroxidases LPR1 and LPR2 (LPRs) were both specifically expressed in the vascular tissues of *Arabidopsis thaliana*, while disruption of both of them increased Fe(II) in the xylem sap and caused excessive Fe deposition in the xylem vessel wall under Fe-sufficient conditions. As a result, a large amount of Fe accumulated in both roots and shoots, hindering plant growth. Moreover, under low-Fe conditions, *LPRs* were preferentially induced in old leaves, but the loss of LPRs increased Fe deposition in the vasculature of older leaves and impeded Fe allocation to younger leaves. Therefore, disruption of both LPRs resulted in severer chlorosis in young leaves under Fe-deficient conditions. Taken together, the oxidation of Fe(II) to Fe(III) by LPRs in the cell wall of vasculature plays an important role in xylem Fe allocation, ensuring healthy Fe homeostasis for normal plant growth.

## Introduction

Iron (Fe) is an essential trace element for plant growth. Due to its redox-active nature under biological conditions, Fe plays a crucial role in metabolic processes such as photosynthesis, respiration, nitrogen fixation, and assimilation (Tagawa and Arnon, 1962; Evans and Russell, 1971; Rotaru and Sinclair, 2009; Balk and Schaedler, 2014). Although total Fe in soil is generally high, the availability of it is greatly affected by environmental factors, such as pH and redox potential (Colombo et al., 2014). Leaf chlorosis of plants due to Fe deficiency is common in alkaline soils where Fe is mostly oxidized and precipitated with low availability for plants (Anderson, 1982). On the contrary, Fe toxicity often occurs in poorly drained soils (under anaerobic conditions) and paddy with low pH, in which Fe(III) is dramatically reduced to Fe(II) (Aung and Masuda, 2020).

Sessile plants have evolved sophisticated regulatory mechanisms to maintain Fe homeostasis under different environmental factors that affect Fe availability (Ravet and Pilon, 2013). So far, many genes involved in Fe uptake and regulation have been identified in the model plant *Arabidopsis thaliana*, such as *ferric reduction oxidase 2* (*FRO2*) (Robinson et al., 1999), Fe^2+^ transporter *iron-regulated transporter 1* (*IRT1*) (Vert et al., 2002), and several basic helix–loop–helix (*bHLH*) transcriptional regulators (Yuan et al., 2005; Wang et al., 2013; Gao et al., 2020). After being absorbed by roots from growth media, the long-distance transport of Fe in the xylem plays important roles in determining shoot Fe nutrition and the systemic signaling of Fe status (White, 2012). As the xylem vessels are composed of dead cells, Fe loading into the xylem is a process from symplast to apoplast (Morris et al., 2018). In *A. thaliana*, Ferroportin 1 (FPN1) (also known as IREG1) is localized on the plasma membrane of pericycle, proposing a possible transporter for Fe(II) loading into the xylem (Morrissey et al., 2009). Unlike Fe(II) loaded into the xylem, Fe(III)–citrate complex was proved to be the main form of the long-distance transport (Rellán-Alvarez et al., 2010; Terzano et al., 2013; Ariga et al., 2014). The citrate for the formation of Fe(III)–citrate in the xylem is secreted by the transporter ferric reductase defective 3 (FRD3) (Green and Rogers, 2004; Durrett et al., 2007; Roschzttardtz et al., 2011). However, to date, little information is available for Fe(II) oxidation in the xylem for the formation of Fe(III)–citrate chelate.

Previous studies in *A. thaliana* have found that an enzyme encoded by *low phosphate root 1 (LPR1)* catalyzes the oxidation of Fe(II) to Fe(III) (Svistoonoff et al., 2007; Muller et al., 2015; Naumann et al., 2022). The LPR1 protein is mainly located in the cell wall matrix (Muller et al., 2015), and it involves in regulating the inhibition of primary root growth in a Fe dose-dependent manner under low phosphate conditions (Ziegler et al., 2016; Heisters, 2019; Wang et al., 2019). However, whether the LPR1 is involved in the other biological processes remains unknown. *Low phosphate root 2 (LPR2)* is the only homologous gene of *LPR1* in *A. thaliana*. Our recent study found that the LPR2 protein is also resided in the cell wall matrix and has ferroxidase activity to catalyze the oxidation of Fe(II) to Fe(III), both of which are very similar to LPR1 (Liu et al., 2022). In view of the Fe(II) oxidation activity of LPR1 and LPR2 (LPRs), these two ferroxidases are likely to play a vital role in plant Fe nutrition, but related studies have not yet reported.

In this study, we highlight the importance of ferroxidases LPR1 and LPR2 in maintaining Fe homeostasis. We found that these two LPRs are mainly expressed in vascular tissues and participate in the oxidation of Fe(II) to Fe(III), thereby promoting the formation of xylem Fe(III)–citrate complexes to avoid excessive Fe sequestration by the vascular cell wall. This process ensures healthy Fe homeostasis for normal growth of plants under either Fe-sufficient or Fe-insufficient conditions. Our finding may provide a basis for improving iron-enriched food crops by biofortification.

## Results

### LPRs are required for normal growth under Fe-sufficient conditions

**FIGURE 1:**
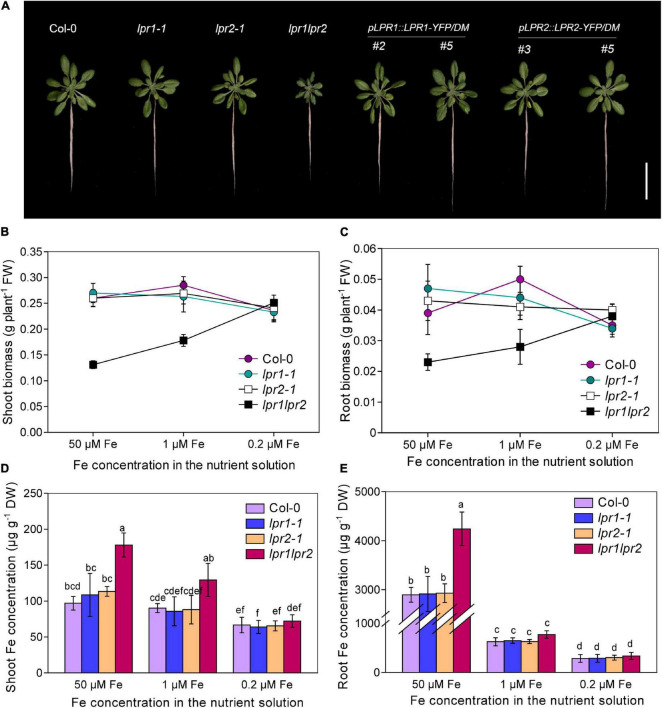
LPRs are required for maintaining healthy Fe status. **(A)** Growth of Col-0, *lpr1-1*, *lpr2-1*, *lpr1lpr2*, and complementation lines (*pLPR1:LPR1-YFP/DM* #2 and #5; *pLPR2:LPR2-YFP/DM* #3 and #5) in Fe-sufficient (50 μM) nutrient solution. DM, *lpr1lpr2* double mutant. **(B,C)** Comparison of shoot fresh biomass and root fresh biomass between Col-0 and *lpr1lpr2* grown under different doses of Fe. **(D,E)** The shoot and root Fe concentrations of the plants grown with different doses of Fe. The plants were precultured in low-Fe (0.2 μM) nutrient solution for 3 weeks and transferred to the nutrient solution with different doses of Fe. Plants were photographed and harvested after treating for 7 days. Values are means ± SD of five replicates. Different letters represent a significant difference at *P* < 0.05 by Tukey’s test. Scale bars 4 cm.

To examine whether the ferroxidases LPR1 and LPR2 are involved in the regulation of Fe nutrition, two T-DNA insertion lines of *LPR1* and *LPR2* (*lpr1-1* and *lpr2-1*) and the double mutant *lpr1lpr2* (generated by crossing *lpr1-1* and *lpr2-1*) were analyzed. When 3-week-old seedlings were transferred to the nutrient solution with sufficient Fe [50 μM Fe(III)–EDTA] supply for 7 days, we found that the single mutants *lpr1-1* and *lpr2-1* did not differ from the wild type; however, the *lpr1lpr2* double mutant exhibited strong stunted and higher chlorophyll levels, with less fresh biomass in shoots and roots (Figures 1A–C and Supplementary Figure 2). We also generated *pLPR1:LPR1-YFP* and *pLPR2:LPR2-YFP* transformants in the background of *lpr1lpr2* double mutant for genetic complementation test. These complementation lines had a higher *LPR1* and *LPR2* expression than wild type did and were able to restore the defects of *lpr1lpr2* double mutant in phenotypes (Figure 1A and Supplementary Figure 1). The above results indicate that *LPR1* and *LPR2* redundantly involve in maintaining normal growth under our growth conditions.

As both LPR1 and LPR2 have been identified as ferroxidases, we determined the concentrations of Fe and other elements in plants. Surprisingly, the Fe concentrations in the shoots and roots were both greatly higher in the *lpr1lpr2* double mutant than in the wild type and single mutants. The Fe concentrations in the shoots and roots of the *lpr1lpr2* were 1.8 and 1.5 times that of the wild type, respectively (Figures 1D,E). We also checked the levels of other nutrients (Supplementary Figure 3); although Zn, Mn, and Cu concentrations also increased significantly, they were not as high as Fe, suggesting that LPRs may be mainly involved in Fe nutrition. Accordingly, the seedlings were subsequently grown with two lower doses of Fe (1 μM and 0.2 μM Fe) to investigate whether the stunted growth of *lpr1lpr2* double mutant is associated with the over-accumulation of Fe. As expected, both lower-Fe-dose treatments improved the growth of *lpr1lpr2* (Figures 1B–E and Supplementary Figure 4). Particularly, the *lpr1lpr2* displayed a similar growth and ionomic phenotypes to that of the wild type under 0.2 μM Fe condition. These results infer that *LPR1* and *LPR2* are functionally redundant and play an important role in maintaining Fe homeostasis for normal growth.

Given that soil pH has a great influence on the availability of Fe (Sarkar and Wynjones, 1982), we also compared the growth of wild type and *lpr1lpr2* in the soils with different pH values. In the moderately acidified soil (pH 5.3), the shoots of *lpr1lpr2* had significantly reduced biomass, but the Fe concentration of *lpr1lpr2* is 4.72 times that of wild type. These differences were greatly minimized when the plants were grown in the soil with pH 6.2 (Supplementary Figure 5). These results provide a further support for that LPR1 and LPR2 are required for maintaining Fe homeostasis in plants.

### LPRs control the behavior of Fe in vascular tissues

**FIGURE 2:**
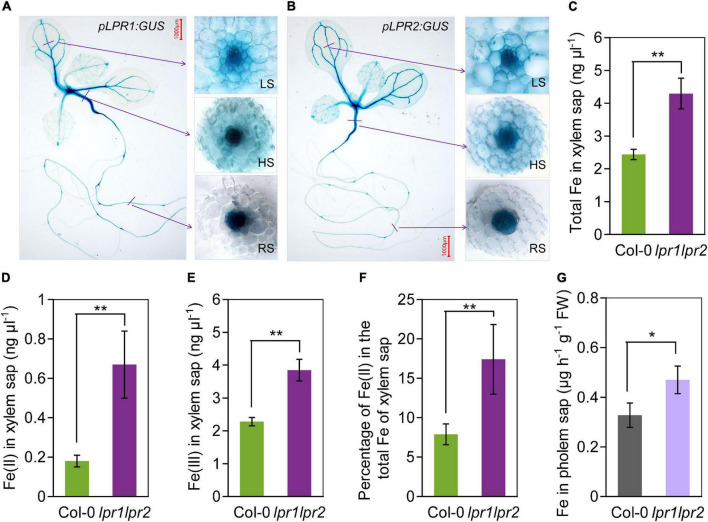
*LPRs* are expressed in vascular and involved in Fe(II) oxidation in xylem. **(A,B)** Histochemical β-glucuronidase (GUS) staining of transformants *pLPR1:GUS*/Col-0 and *pLPR2:GUS*/Col-0. Cross sections of leaf major veins (LS, leaf section), hypocotyls (HS, hypocotyl section), and roots (RS, root section) are in the right inset. The 7-day-old seedlings grown in Fe-sufficient nutrient solution were used for analysis. The total Fe **(C)**, Fe(II) **(D)**, and Fe(III) **(E)** concentration in the xylem saps of Col-0 and *lpr1lpr2*. **(F)** Percentage of Fe(II) in total Fe of xylem saps of Col-0 and *lpr1lpr2*. **(G)** Fe concentration in the phloem saps of Col-0 and *lpr1lpr2*. Plants were precultured in low-Fe (0.2 μM) nutrient solution for 3 weeks and transferred to Fe-sufficient (50 μM) nutrient solution for another 2 weeks, and then, the xylem saps and phloem saps were collected. Values are means ± SD of three replicates. Asterisks indicate a significant difference at *P < 0.05, **P < 0.01, by Tukey’s test. n.s., no significance. Scale bars 1,000 μm.

To identify in which tissues *LPR1* and *LPR2* involve in maintaining Fe homeostasis, we used β-glucuronidase (GUS) as a reporter gene to investigate the expression patterns of *LPR1* and *LPR2*. Histochemical staining of the plants expressing *pLPR1:GUS* and *pLPR2:GUS* revealed that both *LPR1* and *LPR2* were mainly expressed in the vascular tissues of roots, hypocotyls, leaves, lateral roots, stems, sepals, as well as repla to a lesser extent (Figures 2A,B and Supplementary Figure 6). To analyze the vascular tissues in more detail, we examined the expression patterns in the cross sections of roots, hypocotyls, and leaf veins. As shown in the insets of Figures 2A,B, *LPR1* and *LPR2* were mainly expressed in the parenchyma cells surrounding the xylem and phloem, indicating that LPRs may function in the vascular tissues.

The above expression patterns of *LPRs* in vascular tissues promoted us to test LPRs’ roles in Fe long-distance transport in *A. thaliana*. Under our growth condition with sufficient Fe [50 μM Fe(III)–EDTA], the total Fe concentration in the xylem and phloem saps was much higher in *lpr1lpr2* than in wild type (Figures 2C,G), which was consistent with the Fe concentration of shoots (Figures 1D,E). Given that LPRs could oxidize Fe(II) to Fe(III) *in vitro* (Muller et al., 2015; Liu et al., 2022), we subsequently investigated the Fe valence states in the xylem sap based on the ferrozine method (Verschoor and Molot, 2013). Both Fe(II) and Fe(III) concentrations in the xylem saps were markedly increased in *lpr1lpr2* compared with wild type (Figures 2D,E), and the proportion of Fe(II) in the total Fe of xylem sap of *lpr1lpr2* was 2.2-fold the wild type (Figure 2F). These findings suggest that LPRs affect the transport of Fe in the vascular tissues *via* their Fe(II) oxidation activity.

### Shoots and roots of LPRs involve in Fe homeostasis in their corresponding tissues

**FIGURE 3:**
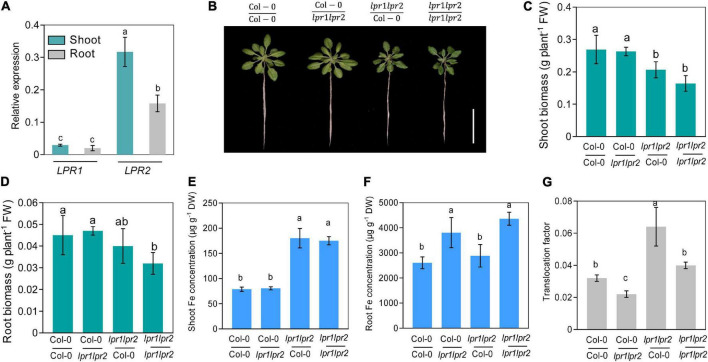
Shoot and root LPRs, respectively, involve in affecting Fe nutrition and growth in their corresponding tissues. **(A)** Gene expression of *low phosphate root 1 (LPR1) and low phosphate root 2 (LPR2)* in shoots and roots. *EF1*α and *UBQ10* were used as the internal control. **(B)** The phenotypes of different grafted plants. Col-0/Col-0 and *lpr1lpr2*/*lpr1lpr2* were self-grafted plants; Col-0/*lpr1lpr2* was grafted plants with Col-0 as shoot scion and *lpr1lpr2* as rootstock; *lpr1lpr2*/Col-0 was grafted plants with *lpr1lpr2* as shoot scion and Col-0 as rootstock. **(C,D)** Comparison of shoot fresh biomass and root fresh biomass between different grafted plants. **(E,F)** Fe concentration in shoots and roots between different grafted plants. **(G)** Translocation factor (TF) of different grafted plants. The grafted plants were photographed and harvested after treating in Fe-sufficient (50 μM) nutrient solution for 7 days. Translocation factor was calculated as shoot Fe/root Fe. Values are means ± SD of four replicates in panel **(A)** and five in panels **(C–G)**. Different letters represent a significant difference at *P* < 0.05 by Tukey’s test. Scale bars 4 cm.

Considering *LPR1* and *LPR2* were expressed in both roots and shoots (Figure 2A), we decided to explore the relative contribution of LPRs in roots versus shoots. We first quantified the *LPR1* and *LPR2* transcripts of wild type. Quantitative real-time PCR (RT-qPCR) assay showed that the expression of *LPR1* was overall lower than that of *LPR2*, and no significant difference was observed between roots and shoots. Nonetheless, a higher *LPR2* expression was identified in shoots (Figure 3A), which prompted us to verify whether LPRs were involved in a shoot-based mechanism; hence, a reciprocal graft experiment under normal Fe growth conditions was employed. As the results shown, grafting wild type shoot scions onto *lpr1lpr2* double mutant rootstocks (i.e., Col-0/*lpr1lpr2*-grafted plants) had normal growth in both shoots and roots (Figures 3B–D). However, the grafted plants with *lpr1lpr2* scions (i.e., *lpr1lpr2*/Col-0- and *lpr1lpr2*/*lpr1lpr2*-grafted plants) grew smaller than the plants with that of wild type. The results indicate that LPRs in shoots rather than in roots are required for ensuring the normal growth phenotype of shoots.

We further measured the Fe level in the grafted plants. The Fe concentration in shoot scions of *lpr1lpr2* double mutant, as expected, was significantly higher than that in wild type (Figure 3E). However, unlike the above growth phenotypes, the Fe concentration of rootstocks was consistent with their genotypes, which was in a shoot-independent manner (Figure 3F). As the translocation factor (TF) can be used to indicate the ability of substance transferred from roots to shoots (Takarina and Pin, 2017), we then calculated the TFs in the grafted plants. An increased TF was found in *lpr1lpr2*/Col-0-grafted plants (Figure 3G), while Col-0/*lpr1lpr2*-grafted plant showed a lower TF than that in other grafted plants. The above results suggest that LPRs in shoots and roots involve in Fe sequestration by their corresponding tissues.

### LPRs are required for Fe deficiency tolerance by preventing Fe allocation to older leaves

**FIGURE 4:**
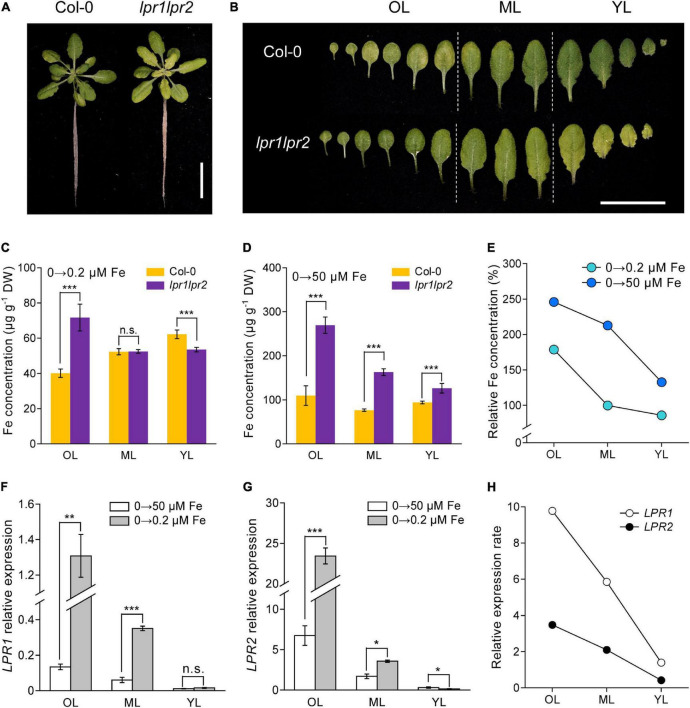
LPRs alleviate Fe deficiency by preventing Fe allocation to old leaves. **(A,B)** Phenotypes of Fe-free pre-grown plants resupplied with 0.2 μM Fe. The Fe concentrations in different leaf positions of Fe-free pre-grown plants resupplied with 0.2 μM Fe **(C)** and 50 μM Fe **(D)**. **(E)** Relative Fe concentration in different leaf positions calculated as *lpr1lpr2* versus Col-0. OL, old leaf; ML, middle leaf; YL, young leaf. **(F,G)** Expression of *low phosphate root 1 (LPR1)* and *low phosphate root 2 (LPR2)* in different leaf positions of the Col-0 plants. *EF1*α and *UBQ10* were used as the internal control. **(H)** Relative expression rates of *LPR1* and *LPR2* in Col-0 calculated as 0.2 μM Fe versus 50 μM Fe treatment. The plants were grown in 0 μM Fe for 7 days and then resupplied with 0.2 or 50 μM Fe for another 5 days. Values are means ± SD of five replicates in **(C,D)** and four in panels **(F,G)**. Asterisks indicate a significant difference at *P < 0.05, **P < 0.01, ***P < 0.001 by Tukey’s test. Scale bars 2 cm.

The action of LPRs in maintaining Fe homeostasis under normal Fe growth conditions raises a question that how LPRs act under Fe-deficient conditions. When seedlings were grown in Fe-free nutrient solution, there was no noticeable difference in chlorosis between wild type and *lpr1lpr2* double mutant (Supplementary Figure 7A). Leaf chlorosis was absent in wild type after 0.2 μM Fe resupply, while *lpr1lpr2* still exhibited obvious yellowish color in the newly expanded leaves (Figure 4A). This appearance was also observed when plants were grown in a soil with alkalization treatment which lowered Fe availability (Supplementary Figure 7B). By comparing the alignment of leaves, we were surprised to find that the color of old leaves of *lpr1lpr2* double mutant was greener than that of wild type (Figure 4B).

The above phenotype was consistent with the results of Fe concentration measured in different ages of leaves: The *lpr1lpr2* had a lower Fe level in young leaf but a higher Fe level in old leaf compared with wild type plants (Figure 4C). We also measured the Fe concentration in different ages of leaves from the plants grown in Fe-free pre-grown plants resupplied with sufficient Fe (50 μM Fe). Under this Fe condition, although the Fe concentration in all leaves of *lpr1lpr2* was higher than wild type, the difference was much greater in old leaves (Figure 4D). The calculation of relative Fe concentration (*lpr1lpr2* vs. wild type) indicated that Fe accumulated preferentially in older leaves of the *lpr1lpr2* double mutant compared with wild type, which was independent of Fe supplementation dose (Figure 4E).

The response of *LPRs* in the wild type to different Fe supplementation doses was then examined. Regardless of the Fe dose, the expression of *LPRs* in old leaves was higher than that in young leaves. It is also worth noting that Fe deficiency upregulated the expression of *LPR1* and *LPR2*, especially in older leaves (Figures 4F–H). The calculation of relative expression of *LRP1* and *LPR2* (0.2 μM Fe vs. 50 μM Fe) showed that Fe deficiency preferentially stimulated these two genes in older leaves. Histochemical GUS staining further confirmed that the expression of *LPRs* in older vascular tissues was preferentially induced under low-Fe conditions (Supplementary Figures 8A,B). Taken together, these data indicate that LPRs may function mainly in older leaves to prevent Fe over-accumulation, which favors the Fe allocation to younger leaves under Fe-deficient conditions.

### LPRs are required for the prevention of abnormal Fe sequestration in vascular tissues

**FIGURE 5:**
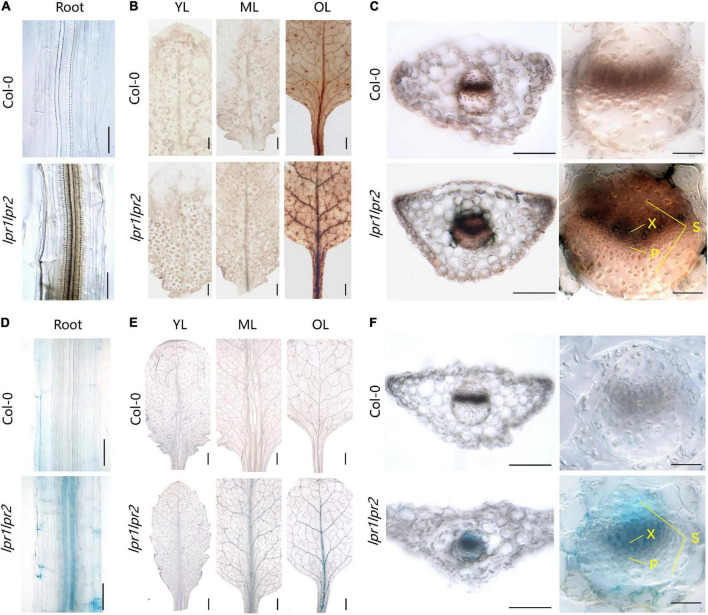
Iron accumulation in the vascular tissues of *lpr1lpr2* double mutant. **(A–C)** Perls/DAB staining of Col-0 and *lpr1lpr2*. **(A)** Close-up views of roots. **(B)** Leaves from different positions. OL, old leaf; ML, middle leaf; YL, young leaf. **(C)** Cross sections of old leaves (left) and close-up views of vascular vessels (right). X, xylem; P, phloem; S, sclerenchyma. **(D–F)** Turnbull staining of Col-0 and *lpr1lpr2*. **(D)** Close-up views of roots. **(E)** Leaves from different positions. **(F)** Cross sections of old leaves and close-up views of vascular vessels. Col-0 and *lpr1lpr2* were precultured in low-Fe (0.2 μM) nutrient solution for 3 weeks and transferred to Fe-sufficient (50 μM) nutrient solution for another 7 days. Scale bars 200 μm in panels (**A,C**, left), (**D,F**, left); 50 μm in panels (**C**, right), (**F**, right); 1,000 μm in panels **(B,E)**.

The above findings led us to investigate the mechanism by which LPRs affect Fe homeostasis in plants. The Perls/DAB (diaminobenzidine) staining was first used to decipher the localization of Fe under normal growth conditions (50 μM Fe). Perls/DAB displayed heavy Fe accumulation in the root stele of *lpr1lpr2* double mutant, whereas such a distribution was not observed in wild type (Figure 5A). In shoots, the vast majority of Fe was located within the vascular bundles of *lpr1lpr2* in old leaves (Figure 5B), which was coincided with the *LPRs* expression. We also compared Fe staining in young and middle leaves, and a slight enhancement of staining color was found in *lpr1lpr2* double mutant versus wild type.

To have a close-up view of Fe accumulation in the vascular bundles, the cross section of old leaves was examined. As shown in Figure 5C, we were surprised to find that Fe was mainly deposited at the periphery of xylem vessel, which is a cell wall matrix and thus coincides with the cell wall localization feature of either LPR1 or LPR2 (Muller et al., 2015; Liu et al., 2022). However, the cells adjacent to the xylem vessel of *lpr1lpr2* also had deeper Fe staining than that of wild type. These data suggest more Fe unloading from the xylem vessel due to LPRs disruption, possibly because the available Fe in the xylem sap of *lpr1lpr2* is higher (Figure 2B). The above notion was also supported by a higher Fe concentration in the phloem of *lpr1lpr2* under sufficient Fe conditions (Figure 2F).

We also performed Perls/DAB staining under 0.2 μM Fe conditions. The Fe deposition in old leaves of *lpr1lpr2* could still be observed in this low-Fe treatment (Supplementary Figure 9A). The greater Fe deposition in the cell wall matrix of xylem vessel could be expected to decrease Fe unloading from the xylem vessel under low-Fe condition. This assumption was confirmed by the observation that the Fe concentration in the phloem of *lpr1lpr2* was less than that of wild type under low-Fe conditions (Supplementary Figure 10).

**FIGURE 6:**
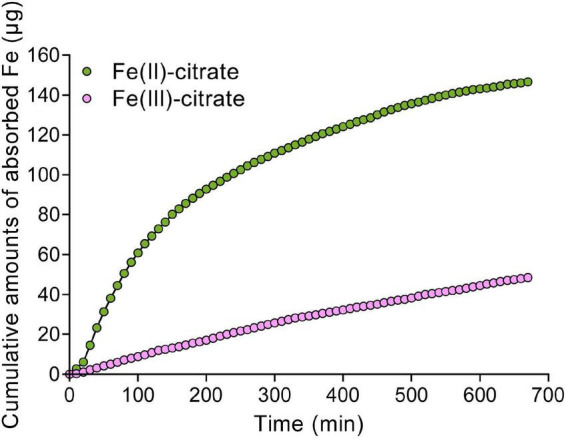
The Fe adsorption kinetics of cell wall for Fe(II)–citrate and Fe(III)–citrate. Ten milligrams of cell wall powder was placed in a 2-ml column. The composition of adsorption solution consisted of 0.1 M sodium acetate (pH 5.0) solution with 50 μM Fe(II)–citrate or Fe(III)–citrate. Then, the residual iron concentration in the collected solution was measured with 2,2-bipyridyl as described in “Materials and methods” section.

Given the Fe(II) oxidation by LPRs, we further tested the distribution characteristics of Fe(II) by Turnbull staining. As expected, Fe(II) was also found to be mainly located in the vascular tissues, particularly in the xylem vessels (Figures 5D–F and Supplementary Figure 9B). This was similar to the Perls/DAB staining above. Then, we asked why the disruption of LPRs results in abnormal Fe sequestration in the cell wall of xylem vessel? We therefore extracted the cell wall of *A. thaliana* to simulate the migration of Fe–citrate in this matrix. The adsorption kinetics analysis reveals that the cell wall had a stronger adsorption capacity for Fe(II)–citrate rather than that for Fe(III)–citrate (Figure 6), providing a reasonable explanation for the above question.

### Disruption of LPRs disturbed the expression of iron-responsive genes

**FIGURE 7:**
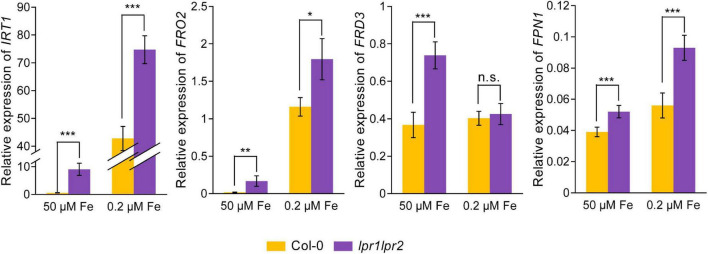
Altered expression of Fe homeostasis-related genes in roots of *lpr1lpr2* double mutant. The expression of *IRT1*, *FRO2*, *FRD3*, and *FPN1* in roots of Col-0 and *lpr1lpr2*. The plants were precultured in low-Fe (0.2 μM) nutrient solution for 3 weeks and then treated with sufficient Fe (50 μM) or low-Fe (0.2 μM) for 4 days. *EF1*α and *UBQ10* were used as the internal control. Values are means ± SD of four replicates. Asterisks indicate a significant difference at *P < 0.05, **P < 0.01, ***P < 0.001 by Tukey’s test.

As Fe concentration in the xylem sap was increased due to the disruption of LPRs, we further investigated the gene expression related to Fe homeostasis by RT-qPCR assay. The results showed that under Fe-sufficient conditions, Fe uptake genes *IRT1* and *FRO2*, as well as iron-loading into xylem genes *FRD3* and *FPN1*, were induced to be significantly higher in the roots of *lpr1lpr2* double mutant, but displayed a lower expression in the wild type (Figure 7). Under low-Fe conditions, the above genes were upregulated in wild type compared with that observed under normal Fe growth conditions, but were still expressed at higher levels in *lpr1lpr2* except *FRD3*. These data exhibited the constitutive expression of Fe deficiency responses in *lpr1lpr2* double mutant, independent of the Fe dose, which is consistent with the higher Fe concentration in *lpr1lpr2* roots and shoots under normal Fe growth conditions (Figures 1D,E). The above results infer that LPRs are also required to maintain the appropriate expression of the genes related to Fe homeostasis in plants, which may be indirectly associated with the role of LPRs in preventing abnormal Fe sequestration in the vascular tissues.

## Discussion

Either Fe deficiency or overload can trigger oxidative damage in plants due to disordered electron transfer. As such, the amount of Fe in plants should be tightly controlled to maintain a healthy Fe status. The apoplastic space accounts for 5% or less plant tissue volume (Loìpez-Millaìn et al., 2000), while such a small apoplastic volume is the main route of Fe transport. Thus, Fe ionic conditions in the apoplast, especially *via* xylem, are of great importance for the Fe deficiency and Fe toxicity (Sattelmacher, 2001). In this study, we revealed that the Fe(II) oxidation by LPRs in the vascular tissue plays an important role in controlling Fe long-distance transport in the xylem, as well as maintaining systemic Fe homeostasis in plants.

As aforementioned, after being absorbed by roots, Fe is loaded into the xylem in the form of Fe(II) (Morrissey et al., 2009). However, unlike Fe(II) loaded into the xylem, Fe(III) was proved to be the main form complexed with citrate (Rellán-Alvarez et al., 2010; Terzano et al., 2013; Ariga et al., 2014). This is probably because Fe(III)–citrate rather than Fe(II)–citrate is able to prevent Fe from precipitation under slightly acidic condition (pH 5.5–6.0) of xylem sap (Curie and Briat, 2003). In addition, the form of Fe(III)–citrate is more beneficial to resist Fenton-mediated oxidative stress than that of Fe(II)–citrate under acidic conditions (Bellapadrona et al., 2010). Therefore, Fe(II) oxidation process is essential after Fe(II) is loaded into the xylem. Previously, cell wall-localized LPR1 and LPR2 were identified as multicopper oxidases (MCO) with Fe(II) oxidation activity (Muller et al., 2015; Liu et al., 2022; Naumann et al., 2022). In this study, GUS staining of transgenic *pLPR1:GUS* and *pLPR2:GUS* plants showed that both *LPR1* and *LPR2* were specifically expressed in the vascular tissues (Figures 2A,B). Furthermore, disruption of *LPRs* resulted in a higher level of Fe(II) in the xylem sap (Figures 2C–F). These findings clearly suggest that LPRs are responsible for, or at least involved in, the Fe(II) oxidation process in the xylem vessels.

Given that Fe(III)–citrate form helps to prevent Fe precipitation in the acidic xylem environment, Fe(II) oxidation by LPRs in the cell wall is expected to affect the movement efficiency of Fe in the xylem. Indeed, grafting experiment revealed that LPRs in shoots and roots involve in Fe homeostasis in their corresponding tissues (Figures 3B–F). Thus, both shoots and roots of LPRs have a significant effect on Fe translocation factor (Figure 3G), which is used to characterize the transport capacity of substances from roots to shoots (Takarina and Pin, 2017). Histological analysis with Fe-specific staining also revealed that disruption of both LPRs resulted in greater Fe deposition in the vascular bundles, particularly in the periphery of xylem vessel (Figure 5). This is probably because the cell wall has a stronger binding capacity for Fe(II)–citrate than Fe(III)–citrate (Figure 6). This can also be explained from a chemical point of view, and citrate has a higher affinity constant for Fe(III) compared with Fe(III) at pH 5.5–6.0 (Sági-Kazár et al., 2022). Besides, Fe(II)–citrate is prone to reoxidize over time and form stable polynuclear complexes of Fe(III)–citrate under moderately acidic conditions (Silva et al., 2009). Taken together, the above findings suggest that disruption of LPRs would be unfavorable for Fe long-distance transport. Previous studies have shown that the loss of FRD3, which impaired long-distance transport of Fe in the xylem, resulted in the disturbance of gene expression of Fe response (Green and Rogers, 2004; Durrett et al., 2007; Roschzttardtz et al., 2011, 2013). Here, we found that disruption of LPRs also disturbed the expression of iron-responsive genes, being similar to that of *frd3* mutant, which provides further support for previous notion. In addition, *lpr1lpr2* double mutant accumulated less Fe as well as exhibited severer chlorosis in young leaves under low-Fe conditions (Figure 4), suggesting a profound effect of the loss of LPRs on Fe long-distance transport.

Given that disruption of LPRs is unfavorable for Fe movement in vasculature, we then analyzed the accumulation of Fe in different leaf positions. The *lpr1lpr2* double mutant accumulated excessive Fe in all leaves, which resulted in a stunted plant growth. One explanation for this is that the loss of LPRs leads to a constitutive Fe deficiency response under Fe-sufficient conditions, including upregulation of *IRT1* and *FRO2*. Thus, *lpr1lpr2* conducted an excessive Fe uptake from the growth medium (Figure 7). Moreover, in the case of excessive Fe uptake, Fe bound to the cell wall may be easily saturated, and the rest of mobile Fe in the xylem still keep a higher level in long-distance transport and subsequent unloading. This assumption was supported by the fact that a higher Fe level was found in the xylem sap, the phloem sap, and the cells adjacent to the xylem of *lpr1lpr2* than wild type under Fe-sufficient conditions (Figures 2C–G, 5C). It is worth noting that LPRs mainly functioned in the vascular tissues of old leaves so as to prevent Fe accumulation under both Fe-sufficient and Fe-insufficient conditions (Figures 4C–E). The spatial features of Fe accumulation coincide with *LPRs* expression patterns, namely that the expression of both *LPR1* and *LPR2* in old leaves was higher than that in young leaves regardless of the Fe dose (Figures 4F–H). Therefore, the increased induction of *LPRs* in old leaves than in young leaves under low-Fe condition may be favorable to prevent Fe distribution to older leaves but to younger leaves, which constitutes a tolerance mechanism to Fe deficiency.

**FIGURE 8:**
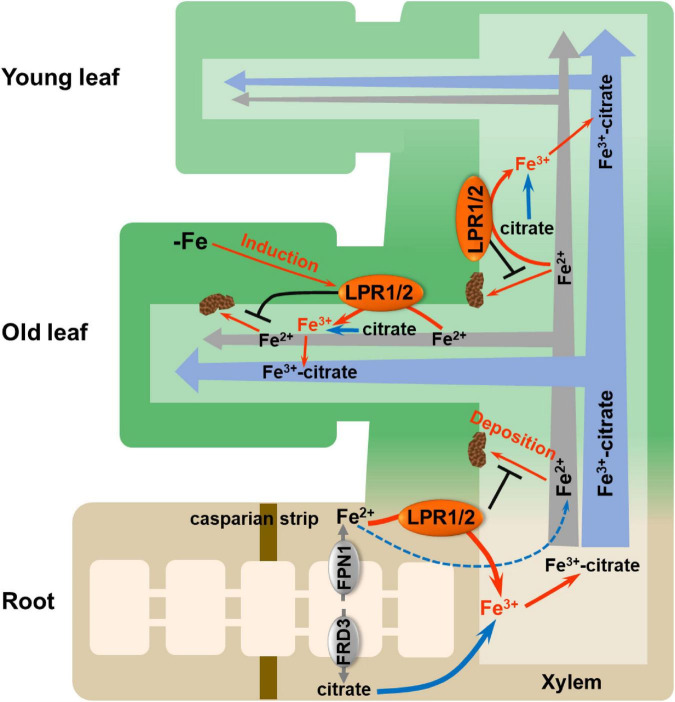
Proposed model for LPRs-mediated Fe(II) oxidation in affecting Fe behavior in xylem. The vascular-located LPRs oxidize Fe(II) to Fe(III) in the cell wall matrix, which facilitates the formation of stable Fe(III)–citrate complex. This process prevents Fe(II) from being bound by the cell wall of xylem. In addition, low-Fe conditions preferentially induced the expression of *LPRs* in the old leaves, which avoids Fe deposition in the xylem cell wall of old leaves and thus favors the allocation of more Fe to younger leaves. LPR1/2 LPR1 and/or LPR2.

Overall, we fill a long-sought blank regarding how Fe(II) is oxidized to Fe(III) in the xylem and highlight the importance of LPRs in maintaining systemic Fe homeostasis (Figure 8). Vascular-located LPRs oxidize Fe(II) to Fe(III), which then binds to citrate to form a stable Fe(III)–citrate complex. This complex helps to prevent Fe from being retained by cell wall, as well as avoid the disturbed gene expression related to Fe nutrition. Furthermore, under low-Fe conditions, the increased expression of *LPRs*, especially in older leaves, avoids Fe deposition in the vascular vessels of old leaves that facilitates Fe migration to younger leaves. Our findings may provide a strategy for improving the efficiency of Fe long-distance transport in plants *via* biotechnological pathways to manipulate the *LPRs* expression in the vascular tissues.

## Materials and methods

### Plant material and growth condition

All plant lines used in this study were in *A. thaliana* ecotype Col-0 background. The mutant seeds of *lpr1-1, lpr2-1*, and *lpr1-1lpr2-1* were previously described (Dong et al., 2017; Liu et al., 2022). The *pLPR1:GUS*/Col-0 transgenic line was a kind gift from Jian-Li Yang (Xu et al., 2020). The seeds were surface-sterilized with 25% NaClO and vernalized at 4°C for 3 days. Then, the seeds were germinated on a nylon mesh and then transferred to the nutrient solution as previously described (Fang et al., 2016). The seedlings were grown under the controlled environment with a 12-h light/12-h dark photoperiod and 100 μmol m^–2^s^–1^ light intensity at 22°C. Considering the *lpr1lpr2* double mutant had developmental defects in Fe-sufficient media, the seedlings were precultured in the nutrient solution with 0.2 μM Fe(III)–EDTA. The other nutrients in the solution were KNO_3_ (1.5 mM), CaCl_2_ (1 mM), NaH_2_PO_4_ (0.5 mM), MgSO_4_ (0.25 mM), (NH_4_)_2_SO_4_ (0.25 mM), H_3_BO_3_ (10 μM), MnSO_4_ (0.5 μM), ZnSO_4_ (0.5 μM), CuSO_4_ (0.1 μM), and (NH_4_)_6_Mo_7_O_24_ (0.1 μM) at pH 5.8. The nutrient solution was refreshed every 4 days. After 21-day preculture, the seedlings were transferred to different doses of Fe(III)–EDTA for further experiments.

For pot experiment, seedlings were germinated on nylon net and then transferred to the peat soil with pH 5.3 (moderately acidified soil) or 6.2 (normal soil) or 7.3 (alkaline soil). The pH of peat soils was adjusted by adding different amounts of CaO. The seedlings were watered weekly with iron-free nutrient solution and harvested after 4 weeks.

### Construction of vectors and generation of transgenic plants

To construct genetic complementation lines, a 5.9-kb genomic DNA fragment of LPR1 and a 4.3-kb genomic DNA fragment of LPR2 (each fragment includes 2.9- and 2.2-kb promoters, respectively) were designed and cloned into the pEarleyGate 101 vector containing a YFP reporter as previously described (Liu et al., 2022). The constructs were transformed into the *Agrobacterium tumefaciens* strain GV3101 and then introduced into *lpr1lpr2* double mutant by the floral dip method. For the construction of the *pLPR2:GUS*/Col-0 transgenic line, a 2.8-kb promoter of *LPR2* was cloned into the pCAMBIA 1,301 vector. The resulting construct was transformed into Col-0. All primers used in this study are listed in Supplementary Table 1. Homozygous T3 transgenic plants were used in subsequent studies.

### Quantitative real-time polymerase chain reaction

Total RNA in various parts of plant was extracted using RNAiso Plus regent (code no. 9109, TaKaRa, China) following the instruction (MacRae, 2007), and the RNA was quantified and assessed for degradation using a micro-spectrophotometer (Nano-400A, Allsheng, China). For real-time PCR (RT-PCR), 800 ng RNA was used to synthesize the first-strand cDNA by HiScript II Q Select RT SuperMix for qPCR (+gDNA wiper) (code no. R223-01, Vazyme, China). For RT-qPCR, the reaction mixture contained 1 μl cDNA template, 0.5 μl forward primer, 0.5 μl reverse primer, 8 μl ddH_2_O, and 10 μl Taq Pro Universal SYBR qPCR Master Mix (code no. Q712-02, Vazyme, China). We performed RT-qPCR analysis on a StepOnePlus Real-Time PCR System (code no. 4376600, Thermo Fisher Scientific, Carlsbad, CA, United States). The specificity of primers was confirmed by melting curve analysis. *EF1*α and *UBQ10* were used as the internal control. The primers used for RT-qPCR are listed in Supplementary Table 1.

### Histochemical β-glucuronidase staining

To analyze GUS activity, the plant materials were stained as described in the study of Ge et al. (2022). In brief, *pLPR1:GUS/*Col-0 and *pLPR2:GUS*/Col-0 plants were incubated in staining buffer (50 mM PBS at pH 7.0, 0.1% Triton X-100, 2 mM K_3_Fe[CN]_6_, 2 mM K_4_[Fe(CN)_6_]⋅3H_2_O, 10 mM Na_2_EDTA⋅2H_2_O, 2 mM X-Gluc) at 37°C overnight. Then, the plants were incubated in 70% ethanol at 37°C for decolorization and optically cleared with chloral hydrate. The stained samples were observed using a microscope (Nikon, Tokyo, Japan).

### Grafting experiment

Grafting experiment was performed as previously described with some modifications (Marsch-Martinez et al., 2013; Ye et al., 2021). The seeds were surface-sterilized and sown on 1/2 MS medium (Coolaber) containing 1% agar and 0.5% sucrose at pH 5.8. After 6 days of germination, seedlings were transversely cut in the middle of the hypocotyl. Afterward, shoot scions and rootstocks were joined on the new plate with the same medium. Ten days later, the adventitious roots were removed, and plants were transferred to hydroponic culture with low-Fe (0.2 μM Fe) nutrient solution for another 7 days of preculture. Finally, the grafted plants were harvested 7 days after transferring to Fe-sufficient (50 μM Fe) nutrient solution.

### Xylem sap collection and detection

To collect the xylem sap, the plants were grown in hydroponic culture with low-Fe nutrient solution for 3 weeks and transferred to Fe-sufficient nutrient solution for another 2 weeks. After rinsing the hypocotyl with ultrapure water, the hypocotyl was transversely cut, and four plants were used as a sample. The xylem sap was collected within 0.5 h with some modifications (Schuler et al., 2012; Chao et al., 2021). The first drop of the xylem sap was discarded, and the subsequent sap was collected in a 1.5-ml tube with 100 μl 1 M sodium acetate (pH 5.0). After collection, the total volume of each sample was recorded, and they were then divided into two equal parts for subsequent determination of ferrous iron and total iron as described (Verschoor and Molot, 2013).

### Collection of phloem exudates and detection

The plants were grown in hydroponic culture for 5 weeks. After rinsing with ultrapure water, eight leaves of each two plants were detached at the petiole. Phloem exudates were collected within 1 h by using EDTA-facilitated method as described (Zhai et al., 2014; Chao et al., 2021). After collection, the fresh weight of leaves was recorded, and the phloem collections were diluted with 1.5 ml of 5% HNO_3_ for subsequent detection by inductively coupled plasma MS (ICP-MS, Thermo Fisher Scientific, United States).

### Elemental analysis

Harvested plants were first rinsed with 5 mM CaCl_2_ for 5 min and then rinsed three times with ultrapure water. Afterward, the samples were dried in an oven at 65°C for 72 h, and the dried weight was recorded. The plant samples were then digested using HNO_3_ at 140°C until the mixture became clear. After dilution with ultrapure water and filtering with a 0.22-μm filter, the Fe and other element concentrations were analyzed by a microwave plasma-atomic emission spectroscope (MP-AES, Agilent Technologies, Santa Clara, CA, United States).

### Histochemical Fe staining

Both Fe(II) and Fe(III) were detected by the Perls/DAB (3,3′-diaminobenzidine tetrahydrochloride) method as described in the study of Roschzttardtz et al. (2013). In brief, plant tissues were rinsed with 2 mM CaSO_4_ and 10 mM EDTA for 5 min and washed three times with ultrapure water. Then, they were fixed in fixation solution [methanol/chloroform/glacial acetic acid (6:3:1)] for 1 h. After rinsing, the materials were incubated in staining solution [2% K_4_Fe(Cn)_6_ and 2% HCl] for 1 h. These samples were then incubated in the prepared solution (0.01 M NaN_3_ and 0.3% H_2_O_2_ in methanol) for 1 h. Finally, the samples were incubated in intensification solution (0.025% DAB, 0.005% H_2_O_2_ in 0.1 M phosphate buffer, pH 7.0) for 5–15 min. For Turnbull staining, the plant samples were incubated for 1 h in 2% K_3_Fe(CN)_6_ and 2% HCl as described (Sperotto et al., 2010). After staining, the samples were imbedded in 4% agarose. Approximately 150-μm cross sections were cut with a vibratome (ZQP-86, Shanghai Zhixin Instrument, Shanghai, China). The stained samples were observed using a microscope (Nikon, Tokyo, Japan).

### Adsorption kinetics analysis

The cell wall was extracted as described (Zhu et al., 2020). Then, the Fe adsorption kinetics of cell wall were analyzed as described earlier (Jin et al., 2007). In brief, 10 mg dried cell wall powder was loaded into a 2-ml column with a filter at both ends. The composition of adsorption solution consisted of 0.1 m sodium acetate (pH 5.0) solution with 50 μM Fe(II)–citrate [prepared from Fe(NH_4_)_2_⋅(SO_4_)_2_⋅6H_2_O and citrate in equimolar proportions] or 50 μM Fe(III)–citrate (prepared from FeCl_3_ and citrate in equimolar proportions). The solution was pumped by a peristaltic pump at a speed of 4.4 ml min^–1^ passing through the column. Then, the collected solution was reduced with 2% ascorbic acid and determined by 0.2% 2,2-bipyridyl at 520 nm (Heaney and Davison, 1977).

### Statistical analysis

The data were analyzed by one-way and two-way analysis of variance (ANOVA) with Tukey’s test using SPSS Statistics version 20.0. A *p*-value <0.05 was considered statistically significant.

## Data availability statement

The original contributions presented in this study are included in the article/Supplementary material, further inquiries can be directed to the corresponding authors.

## Author contributions

Q-YZ and C-WJ conceived the project, interpreted the data, generated the figures, and wrote the manuscript. C-WJ and Y-XZ supervised the experiments. Q-YZ performed the majority of the experiments. YW, X-XL, J-YY, MZ, X-TJ, W-JH, W-XD, and CH assisted in performing the experiments. All authors contributed to the article and approved the submitted version.

## References

[B1] Anderson W. B. (1982). Diagnosis and correction of iron deficiency in field crops – an overview. *J. Plant Nutr.* 5 785–795.

[B2] Ariga T. Hazama K. Yanagisawa S. Yoneyama T. (2014). Chemical forms of iron in xylem sap from graminaceous and non-graminaceous plants. *Soil Sci. Plant Nutr.* 60 460–469.

[B3] Aung M. S. Masuda H. (2020). How does rice defend against excess iron?: Physiological and molecular mechanisms. *Front. Plant Sci.* 11:1102. 10.3389/fpls.2020.01102 32849682 PMC7426474

[B4] Balk J. Schaedler T. A. (2014). Iron cofactor assembly in plants. *Annu. Rev. Plant Biol.* 65 125–153. 24498975 10.1146/annurev-arplant-050213-035759

[B5] Bellapadrona G. Ardini M. Ceci P. Stefanini S. Chiancone E. (2010). Dps proteins prevent Fenton-mediated oxidative damage by trapping hydroxyl radicals within the protein shell. *Free Radic. Biol. Med.* 48 292–297. 10.1016/j.freeradbiomed.2009.10.053 19892013

[B6] Chao Z. F. Wang Y. L. Chen Y. Y. Zhang C. Y. Wang P. Y. Song T. (2021). NPF transporters in synaptic-like vesicles control delivery of iron and copper to seeds. *Sci. Adv.* 7:eabh2450. 10.1126/sciadv.abh2450 34516912 PMC8442890

[B7] Colombo C. Palumbo G. He J.-Z. Pinton R. Cesco S. (2014). Review on iron availability in soil: Interaction of Fe minerals, plants, and microbes. *J. Soils Sediments* 14 538–548.

[B8] Curie C. Briat J. F. (2003). Iron transport and signaling in plants. *Annu. Rev. Plant Biol.* 54 183–206. 14509968 10.1146/annurev.arplant.54.031902.135018

[B9] Dong J. S. Pineros M. A. Li X. X. Yang H. B. Liu Y. Murphy A. S. (2017). An *Arabidopsis* ABC transporter mediates phosphate deficiency-induced remodeling of root architecture by modulating iron homeostasis in roots. *Mol. Plant* 10 244–259. 10.1016/j.molp.2016.11.001 27847325

[B10] Durrett T. P. Gassmann W. Rogers E. E. (2007). The FRD3-mediated efflux of citrate into the root vasculature is necessary for efficient iron translocation. *Plant Physiol.* 144 197–205. 10.1104/pp.107.097162 17351051 PMC1913786

[B11] Evans H. J. Russell S. A. (1971). “Physiological chemistry of symbiotic nitrogen fixation by legumes,” in *The chemistry and biochemistry of nitrogen fixation*, ed. Postgate J. R. (Boston, MA: Springer), 191–244.

[B12] Fang X. Z. Tian W. H. Liu X. X. Lin X. Y. Jin C. W. Zheng S. J. (2016). Alleviation of proton toxicity by nitrate uptake specifically depends on nitrate transporter 1.1 in *Arabidopsis*. *New Phytol.* 211 149–158. 10.1111/nph.13892 26864608

[B13] Gao F. Robe K. Bettembourg M. Navarro N. Rofidal V. Santoni V. (2020). The transcription factor bHLH121 interacts with bHLH105 (ILR3) and its closest homologs to regulate iron homeostasis in Arabidopsis. *Plant Cell* 32, 508–524. 31776233 10.1105/tpc.19.00541 PMC7008485

[B14] Ge H. Wang Y. Chen J. Zhang B. Chen R. Lan W. (2022). An *Arabidopsis* vasculature distributed metal tolerance protein facilitates xylem magnesium diffusion to shoots under high-magnesium environments. *J. Integr. Plant Biol.* 64 166–182. 10.1111/jipb.13187 34761874

[B15] Green L. S. Rogers E. E. (2004). FRD3 controls iron localization in *Arabidopsis*. *Plant Physiol.* 136 2523–2531. 10.1104/pp.104.045633 15310833 PMC523319

[B16] Heaney S. I. Davison W. (1977). Determination of ferrous iron in natural-waters with 2,2’ bipyridyl. *Limnol. Oceanogr.* 22 753–760.

[B17] Heisters M. (2019). *Characterization of the multicopper oxidase LPR1 and the P5-type ATPase PDR2 and their roles in the phosphate starvation response of Arabidopsis thaliana*. Doctoral dissertation. Halle (Saale): Martin-Luther-Universität Halle-Wittenberg.

[B18] Jin C. W. You G. Y. He Y. F. Tang C. X. Wu P. Zheng S. J. (2007). Iron deficiency-induced secretion of phenolics facilitates the reutilization of root apoplastic iron in red clover. *Plant Physiol.* 144 278–285. 10.1104/pp.107.095794 17369430 PMC1913808

[B19] Liu X. X. Zhang H. H. Zhu Q. Y. Ye J. Y. Zhu Y. X. Jing X. T. (2022). Phloem iron remodels root development in response to ammonium as the major nitrogen source. *Nat. Commun.* 13:561. 10.1038/s41467-022-28261-4 35091578 PMC8799741

[B20] Loìpez-Millaìn A. F. Morales F. n. Abadıìa A. n. Abadıìa J. (2000). Effects of iron deficiency on the composition of the leaf apoplastic fluid and xylem sap in sugar beet. Implications for iron and carbon transport. *Plant Physiol.* 124 873–884. 10.1104/pp.124.2.873 11027735 PMC59191

[B21] MacRae E. (2007). “Extraction of Plant RNA,” in *Protocols for nucleic acid analysis by nonradioactive probes*, eds Hilario E. Mackay J. (Totowa, NJ: Humana Press), 15–24.

[B22] Marsch-Martinez N. Franken J. Gonzalez-Aguilera K. L. de Folter S. Angenent G. Alvarez-Buylla E. R. (2013). An efficient flat-surface collar-free grafting method for *Arabidopsis thaliana* seedlings. *Plant Methods* 9:14. 10.1186/1746-4811-9-14 23641687 PMC3668283

[B23] Morris H. Plavcová L. Gorai M. Klepsch M. M. Kotowska M. Jochen Schenk H. (2018). Vessel-associated cells in angiosperm xylem: Highly specialized living cells at the symplast–apoplast boundary. *Am. J. Bot.* 105 151–160. 10.1002/ajb2.1030 29578292

[B24] Morrissey J. Baxter I. R. Lee J. Li L. Lahner B. Grotz N. (2009). The ferroportin metal efflux proteins function in iron and cobalt homeostasis in *Arabidopsis*. *Plant Cell* 21 3326–3338. 10.1105/tpc.109.069401 19861554 PMC2782287

[B25] Muller J. Toev T. Heisters M. Teller J. Moore K. L. Hause G. (2015). Iron-dependent Callose deposition adjusts root meristem maintenance to phosphate availability. *Dev. Cell* 33 216–230. 10.1016/j.devcel.2015.02.007 25898169

[B26] Naumann C. Heisters M. Brandt W. Janitza P. Alfs C. Tang N. (2022). Bacterial-type ferroxidase tunes iron-dependent phosphate sensing during *Arabidopsis* root development. *Curr. Biol.* 32 2189–2205. 10.1016/j.cub.2022.04.005 35472311 PMC9168544

[B27] Ravet K. Pilon M. (2013). Copper and iron homeostasis in plants: The challenges of oxidative stress. *Antioxid. Redox Signal.* 19 919–932. 23199018 10.1089/ars.2012.5084 PMC3763233

[B28] Rellán-Alvarez R. Giner-Martínez-Sierra J. Orduna J. Orera I. Rodríguez-Castrillón J. A. García-Alonso J. I. (2010). Identification of a tri-iron(III), tri-citrate complex in the xylem sap of iron-deficient tomato resupplied with iron: New insights into plant iron long-distance transport. *Plant Cell Physiol.* 51 91–102. 10.1093/pcp/pcp170 19942594

[B29] Robinson N. J. Procter C. M. Connolly E. L. Guerinot M. L. (1999). A ferric-chelate reductase for iron uptake from soils. *Nature* 397 694–700. 10067892 10.1038/17800

[B30] Roschzttardtz H. Conejero G. Divol F. Alcon C. Verdeil J. L. Curie C. (2013). New insights into Fe localization in plant tissues. *Front. Plant Sci.* 4:350. 10.3389/fpls.2013.00350 24046774 PMC3764369

[B31] Roschzttardtz H. Seguela-Arnaud M. Briat J. F. Vert G. Curie C. (2011). The FRD3 citrate effluxer promotes iron nutrition between symplastically disconnected tissues throughout *Arabidopsis* development. *Plant Cell* 23 2725–2737. 10.1105/tpc.111.088088 21742986 PMC3226209

[B32] Rotaru V. Sinclair T. R. (2009). Interactive influence of phosphorus and iron on nitrogen fixation by soybean. *Environ. Exp. Bot.* 66 94–99.

[B33] Sági-Kazár M. Solymosi K. Solti Á (2022). Iron in leaves: Chemical forms, signalling, and in-cell distribution. *J. Exp. Bot.* 73 1717–1734. 10.1093/jxb/erac030 35104334 PMC9486929

[B34] Sarkar A. N. Wynjones R. G. (1982). Effect of rhizosphere pH on the availability and uptake of Fe, Mn and Zn. *Plant Soil* 66 361–372.

[B35] Sattelmacher B. (2001). The apoplast and its significance for plant mineral nutrition. *New Phytol.* 149 167–192. 33874640 10.1046/j.1469-8137.2001.00034.x

[B36] Schuler M. Rellan-Alvarez R. Fink-Straube C. Abadia J. Bauer P. (2012). Nicotianamine functions in the Phloem-based transport of iron to sink organs, in pollen development and pollen tube growth in *Arabidopsis*. *Plant Cell* 24 2380–2400. 10.1105/tpc.112.099077 22706286 PMC3406910

[B37] Silva A. M. Kong X. Parkin M. C. Cammack R. Hider R. C. (2009). Iron(III) citrate speciation in aqueous solution. *Dalton Trans.* 40 8616–8625. 10.1039/b910970f 19809738

[B38] Sperotto R. A. Boff T. Duarte G. L. Santos L. S. Grusak M. A. Fett J. P. (2010). Identification of putative target genes to manipulate Fe and Zn concentrations in rice grains. *J. Plant Physiol.* 167 1500–1506. 10.1016/j.jplph.2010.05.003 20580124

[B39] Svistoonoff S. Creff A. Reymond M. Sigoillot-Claude C. Ricaud L. Blanchet A. (2007). Root tip contact with low-phosphate media reprograms plant root architecture. *Nat. Genet.* 39 792–796. 17496893 10.1038/ng2041

[B40] Tagawa K. Arnon D. I. (1962). Ferredoxins as electron carriers in photosynthesis and in biological production and consumption of hydrogen gas. *Nature* 195 537–543. 10.1038/195537a0 14039612

[B41] Takarina N. D. Pin T. G. (2017). Bioconcentration factor (BCF) and translocation factor (eTF) of heavy metals in mangrove trees of blanakan fish farm. *Makara J. Sci.* 21 78–82.

[B42] Terzano R. Mimmo T. Vekemans B. Vincze L. Falkenberg G. Tomasi N. (2013). Iron (Fe) speciation in xylem sap by XANES at a high brilliant synchrotron X-ray source: Opportunities and limitations. *Anal. Bioanal. Chem.* 405 5411–5419. 10.1007/s00216-013-6959-1 23609785

[B43] Verschoor M. J. Molot L. A. (2013). A comparison of three colorimetric methods of ferrous and total reactive iron measurement in freshwaters. *Limnol. Oceanogr. Methods* 11 113–125.

[B44] Vert G. Grotz N. Dedaldechamp F. Gaymard F. Guerinot M. L. Briat J. F. (2002). IRT1, an *Arabidopsis* transporter essential for iron uptake from the soil and for plant growth. *Plant Cell* 14 1223–1233. 12084823 10.1105/tpc.001388 PMC150776

[B45] Wang N. Cui Y. Liu Y. Fan H. Du J. Huang Z. (2013). Requirement and functional redundancy of Ib subgroup bHLH proteins for iron deficiency responses and uptake in Arabidopsis thaliana. *Mol. Plant* 6, 503–513. 10.1093/mp/sss089 22983953

[B46] Wang X. Wang Z. Zheng Z. Dong J. Song L. Sui L. (2019). Genetic dissection of fe-dependent signaling in root developmental responses to phosphate deficiency. *Plant Physiol.* 179 300–316. 10.1104/pp.18.00907 30420567 PMC6324241

[B47] White P. J. (2012). “Long-distance transport in the xylem and phloem,” in *Marschner’s mineral nutrition of higher plants*, ed. Marschner P. (San Diego, CA: Academic Press), 49–70.

[B48] Xu J. M. Wang Z. Q. Wang J. Y. Li P. F. Jin J. F. Chen W. W. (2020). Low phosphate represses histone deacetylase complex1 to regulate root system architecture remodeling in *Arabidopsis*. *New Phytol.* 225 1732–1745. 10.1111/nph.16264 31608986

[B49] Ye J. Y. Tian W. H. Zhou M. Zhu Q. Y. Du W. X. Jin C. W. (2021). Improved plant nitrate status involves in flowering induction by extended photoperiod. *Front. Plant Sci.* 12:629857. 10.3389/fpls.2021.629857 33643357 PMC7907640

[B50] Yuan Y. X. Zhang J. Wang D. W. Ling H. Q. (2005). AtbHLH29 of Arabidopsis thaliana is a functional ortholog of tomato FER involved in controlling iron acquisition in strategy I plants. *Cell Res.* 15, 613–621. 10.1038/sj.cr.7290331 16117851

[B51] Zhai Z. Gayomba S. R. Jung H. I. Vimalakumari N. K. Piñeros M. Craft E. (2014). OPT3 is a phloem-specific iron transporter that is essential for systemic iron signaling and redistribution of iron and cadmium in *Arabidopsis*. *Plant Cell* 26 2249–2264. 10.1105/tpc.114.123737 24867923 PMC4079381

[B52] Zhu X. F. Wu Q. Meng Y. T. Tao Y. Shen R. F. (2020). AtHAP5A regulates iron translocation in iron-deficient *Arabidopsis thaliana*. *J. Integr. Plant Biol.* 62 1910–1924. 10.1111/jipb.12984 33405355

[B53] Ziegler J. Schmidt S. Chutia R. Muller J. Bottcher C. Strehmel N. (2016). Non-targeted profiling of semi-polar metabolites in *Arabidopsis* root exudates uncovers a role for coumarin secretion and lignification during the local response to phosphate limitation. *J. Exp. Bot.* 67 1421–1432. 10.1093/jxb/erv539 26685189 PMC4762384

