# Supplementary material for: The ferroxidases are critical for Fe(II) oxidation in xylem to ensure a healthy Fe allocation in Arabidopsis thaliana
Source: Front Plant Sci. 2022 Aug 17;13:958984. doi: 10.3389/fpls.2022.958984 (PMC9428407; doi:10.3389/fpls.2022.958984)
Supplement: Supplementary file 1 [file Data_Sheet_1.PDF]

Supplementary Material

1 Supplementary Figures and Tables

1.1 Supplementary Figures

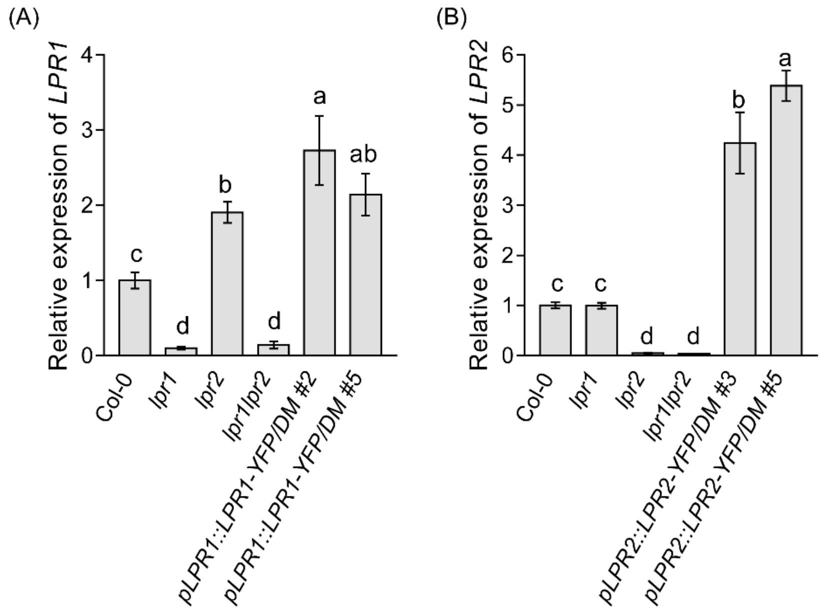

Figure S1. Expression of *LPR1* and *LPR2* in complementation lines. Comparison of the expression level of *LPR1* **(A)** and *LPR2* **(B)** in *pLPR1::LPR1-YFP*/DM and *pLPR2::LPR2-YFP*/DM transgenic lines. 7-day-old seedlings were used for analysis. DM, *lpr1lpr2* double mutant. *EF1α* and *UBQ10* were used as the internal control. Relative expression of Col-0 was set to 1. Values are means±SD of four replicates. Different letters represent a significant difference at $P < 0.05$ by Tukey's test.

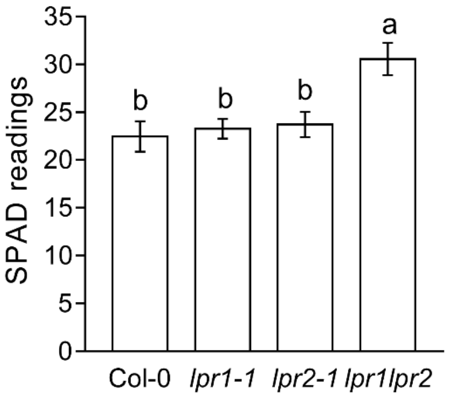

Figure S2. Relative levels of chlorophyll in Col-0 and *lpr1lpr2*. The relative chlorophyll level (SPAD reading) of Col-0 and *lpr1lpr2* were measured by a chlorophyll meter after treating in Fe-sufficient (50 μM) nutrient solution for 7 days. Values are means±SD of eleven replicates. Different letters represent a significant difference at $P < 0.05$ by Tukey's test.

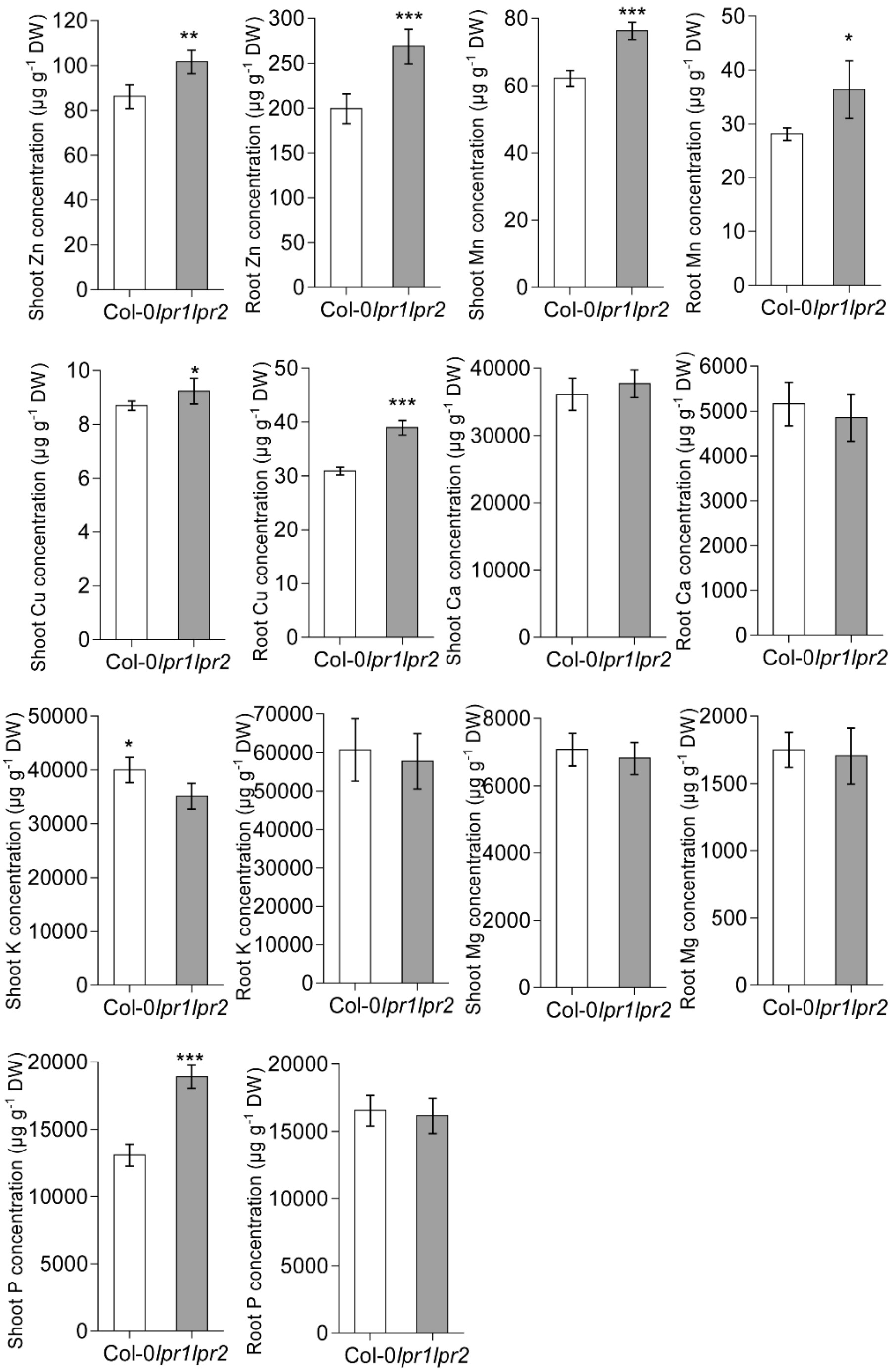

Figure S3. Concentrations of nutrient elements between Col-0 and *lpr1lpr2*. Col-0 and *lpr1lpr2* were precultured in low-Fe nutrient (0.2 μM) solution for 3 weeks and transferred to Fe-sufficient (50 μM) nutrient solution for another 7 days. The concentrations of Mn, Cu, Zn, K, Ca, Mg, P were detected by MP-AES. Asterisks indicate significant difference at *P < 0.05, **P < 0.01, ***P < 0.001 by Tukey's test.

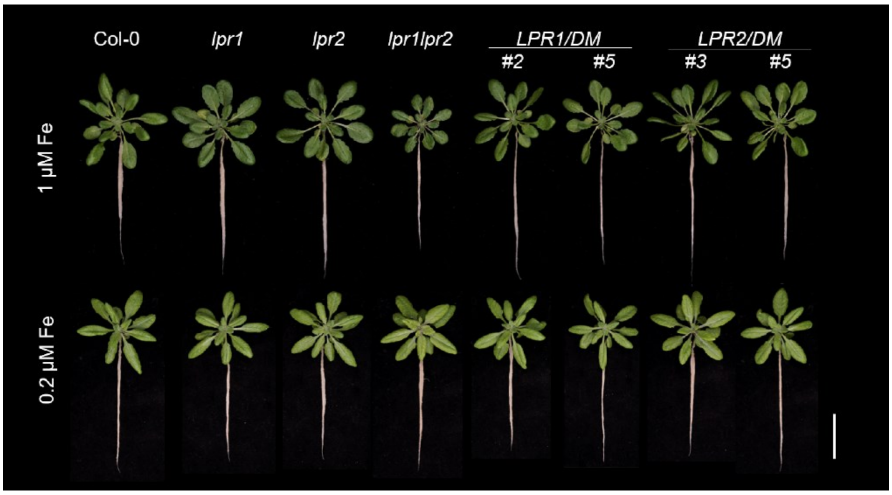

Figure S4. Growth of plants under reduced doses of Fe. Col-0, *lpr1-1, lpr2-1, lpr1lpr2,* and complementation lines (*pLPR1::LPR1-YFP*/DM #2 and #5; *pLPR2::LPR2-YFP*/DM #3 and #5) were precultured in low-Fe nutrient solution for 3 weeks and transferred to 1 μM and 0.2 μM Fe nutrient solution for another 7 days. DM, *lpr1lpr2* double mutant. Scale bars, 4 cm.

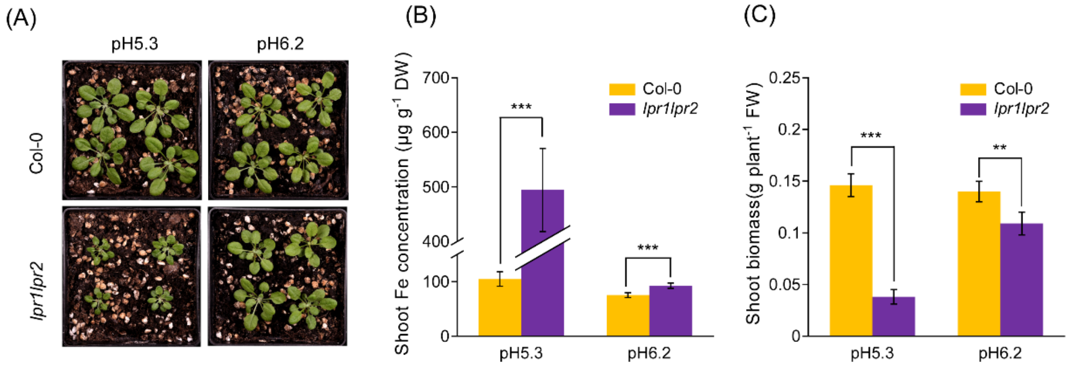

Figure S5. Growth of Col-0 and *lpr1lpr2* in pot experiment. Col-0 and *lpr1lpr2* were germinated on nylon net, and then transferred to soils with different pH (pH 5.3-7.3). Plants were photographed and harvested after treating for 4 weeks. **(A)** Phenotypes of Col-0 and *lpr1lpr2* in pot experiment. **(B)** Comparison of shoot fresh biomass between Col-0 and *lpr1lpr2*. **(C)** Shoot Fe concentrations. Values are means±SD of five replicates. Asterisks indicate significant difference at *P < 0.05, **P < 0.01, ***P < 0.001 by Tukey's test.

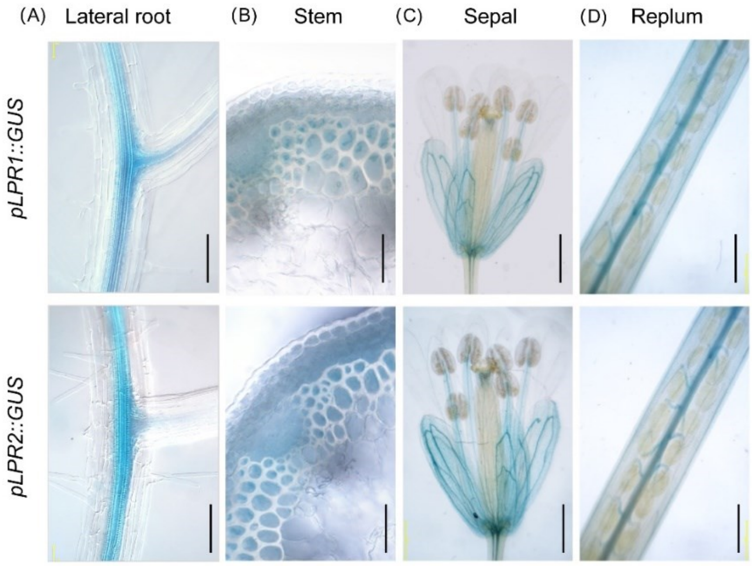

Figure S6. Histochemical GUS staining of *pLPR1::GUS*/Col-0 and *pLPR2::GUS*/Col-0 in different parts. GUS activities of *pLPR1::GUS*/Col-0 and *pLPR2::GUS*/Col-0 in lateral roots **(A)**, stems **(B)**, sepals **(C)**, and repla **(D)**. Scale bars, 100 μm in A, B; 500 μm in C, D.

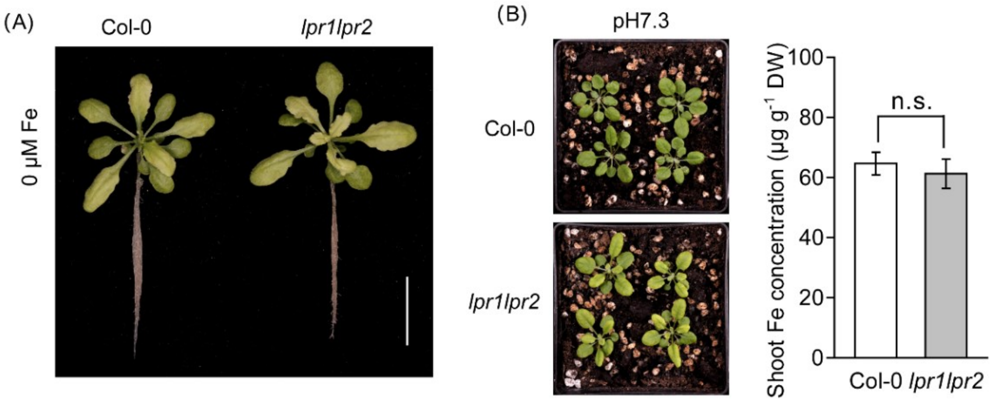

Figure S7. Growth of Col-0 and *lpr1lpr2* under Fe-deficient conditions. (A) Phenotypes of Col-0 and *lpr1lpr2* in Fe-free nutrient solution. Plants were precultured in low-Fe nutrient solution for 3 weeks and transferred to 0 Fe nutrient solution for 7 days. Scale bars, 2 cm. **(B)** Phenotypes of Col-0 and *lpr1lpr2* in alkaline soil. **(C)** Shoot Fe concentration in alkaline soil. Col-0 and *lpr1lpr2* were germinated on nylon net, and transferred to alkaline soil for 4 weeks. Values are means±SD of five replicates. Asterisks indicate significant difference at *P < 0.05, **P < 0.01, ***P < 0.001 by Tukey's test.

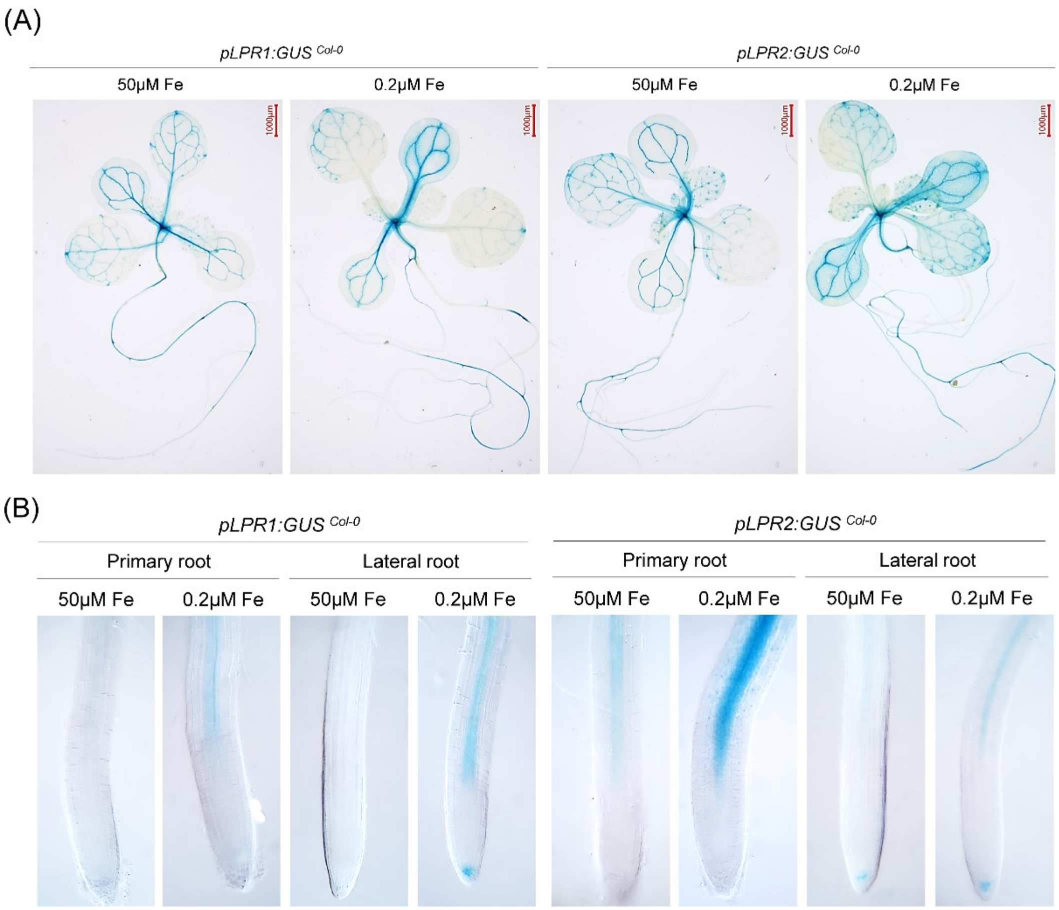

Figure S8. GUS activities of *pLPR1::GUS*/Col-0 and *pLPR2::GUS*/Col-0 in different doses of Fe supplementation. 7-day-old seedlings germinated on nylon mesh were treated with 0.2 µM Fe or 50 µM Fe for 3 days for analysis. **(A)** GUS staining for the whole plant. Scale bars, 1000 µm. **(B)** Close-up views of primary roots and lateral roots. Scale bars, 1000 µm in A; 100 µm in B.

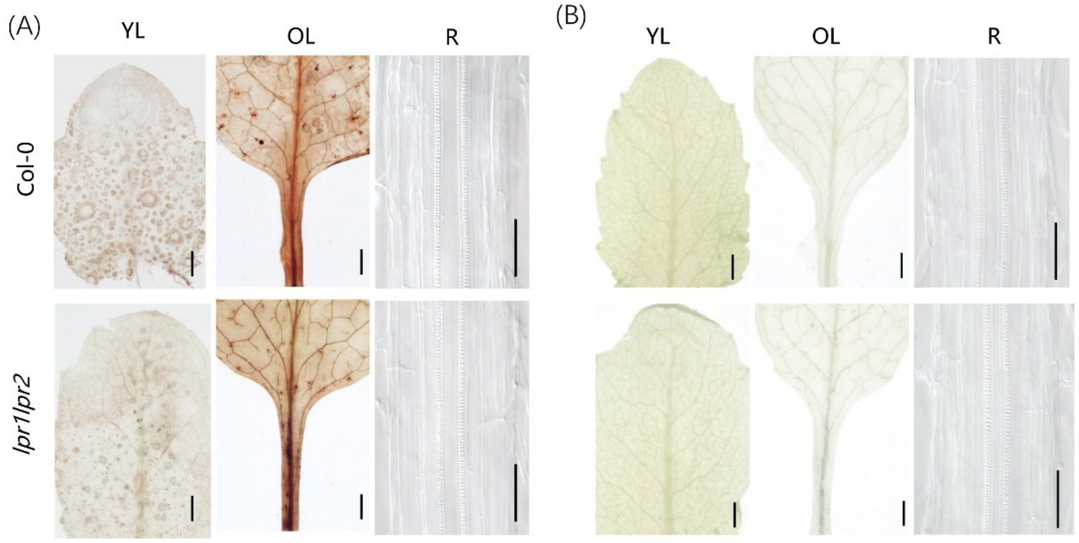

Figure S9. Fe staining under low-Fe conditions. Col-0 and *lpr1lpr2* were cultured in low-Fe (0.2 μM) nutrient solution for 4 weeks and then Fe staining was performed. (A) Perls/DAB staining for leaves from different positions. (B) Turnbull staining for leaves from different positions. OL, old leaf; YL, young leaf. Scale bars, 1000 μm in YL and OL; 200 μm in R.

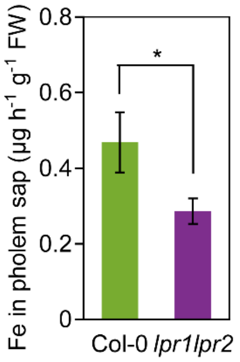

Figure S10. Fe concentration in phloem sap under low-Fe condition. 3-week-old plants precultured in 0.2 µM Fe nutrient solution and then transferred to 0 µM Fe nutrient solution for 1 week, and resupplied 0.2 µM Fe for 5 days. Since then, the phloem saps were collected and analyzed. Values are means±SD of three replicates. Asterisks indicate significant difference at *P < 0.05, **P < 0.01, ***P < 0.001 by Tukey's test.

1.2 Supplementary Tables

Primers used in this study

**Primer**	**Sequence (5'→3')**	**Purpose**
lpr1-1 LP	CTCATCGCCAGTAGGTAGCTG	Genotyping for *lpr1-1* (SALK_016297)
lpr1-1 RP	ACTCATGGGTGTGAACCAAAG	
lpr2-1 LP	CATAGCCTGGCTCTTGAGTTG	Genotyping for *lpr2-1* (SALK_091930)
lpr2-1 RP	GTCATAGCTCAGTCGAATCGC	
LBb1.3	ATTTTGCCGATTTCGGAAC	Genotyping PCR
pLPR1::LPR1-YFP-F	cacagatggttagagaggcctAGATGCTTTTGTTTGTCTTGGTTCT	Construction of *pLPR1::LPR1-YFP*
pLPR1::LPR1-YFP-R	ctcgcccttgctcaccctaggAATGATGACCTTAAGCGGCCT	
pLPR2::LPR2-YFP-F	cacagatggttagagaggcctTCAATGTGTTTAATATCAAAGTATTTTCG	Construction of *pLPR2::LPR2-YFP*
pLPR2::LPR2-YFP-R	ctcgcccttgctcaccctaggTAGCACCATTGCAAAGGGCC	
pLPR2::GUS-F	tatgaccatgattacgaattcTGCAAATTATTATATTAGTGGAATTAATTGA	Construction of *pLPR2::GUS*
pLPR2::GUS-R	ttaccctcagatctaccatggGTCTTTGTCCTTCACGAGCTGTC	
LPR1 qPCR-F	TGGTATCATCAAACCAGCTTCT	qPCR of *LPR1*
LPR1 qPCR-R	GATTTAGGAAGGTGATTTCGCC	
LPR2 qPCR-F	CCGGGACATGTAACGAAGATAC	qPCR of *LPR2*
LPR2 qPCR-R	GGGCCTCATCATCATATTGTCTTC	
EF1α qPCR-F	TGAGCACGCTCTTCTTGCTTTCA	qPCR of *EF1α*
EF1α qPCR-R	GGTGGTGGCATCCATCTTGTTACA	
UBQ10 qPCR-F	ACCCTAACGGGAAAGACGA	qPCR of *UBQ10*
UBQ10 qPCR-R	GGAGCCTGAGAACAAGATGAA	
IRT1 qPCR-F	AAGCTTTGATCACGGTTGG	qPCR of *IRT1*
IRT1 qPCR-R	TTAGGTCCCATGAACTCCG	
FRO2 qPCR-F	GATCGAAAAAAGCAATAACGGTGGTT	qPCR of *FRO2*
FRO2 qPCR-R	GATGTGGCAACCACTTGGTTCGATA	
FRD3 qPCR-F	TTTTGTCGGGCGTTTAGG	qPCR of *FRD3*
FRD3 qPCR-R	TTGCTGTGGCTGGTTGGT	
FPN1 qPCR-F	ATATCATCGGTATAGGCAGGGGA	qPCR of *FPN1*
FPN1 qPCR-R	CGGATTCTGAAACAAGATCCTGC	
